# Safety and effectiveness of a new minimally invasive glaucoma surgery namely trabeculotome tunneling trabeculoplasty in primary open-angle glaucoma

**DOI:** 10.3389/fmed.2025.1641952

**Published:** 2025-09-10

**Authors:** Suzhen Wang, Qin Qiu, Yu He, Hanying Fan, Lin Jing, Liuzhi Zeng, Ningli Wang

**Affiliations:** ^1^Department of Ophthalmology, Chengdu Integrated TCM and Western Medicine Hospital, Chengdu, China; ^2^Beijing Tongren Eye Center, Beijing Tongren Hospital, Capital Medical University, Beijing, China

**Keywords:** primary open-angle glaucoma, minimally invasive glaucoma surgery, intraocular pressure, trabeculotome tunnelling trabeculoplasty, surgical success rate

## Abstract

**Aim:**

This study aimed to report the preliminary surgical outcomes of a new minimally invasive glaucoma surgery (MIGS), namely trabeculotome tunneling trabeculoplasty (3T) in primary open-angle glaucoma (POAG).

**Methods:**

This retrospective observational study with prospective follow-up included POAG patients who underwent 3T surgery at Chengdu First People’s Hospital between December 2022 and June 2024. Postoperative follow-up was conducted at 1 day, 1 week, 1 month, 3 months, 6 months, and 12 months. Evaluations included intraocular pressure (IOP), number of medications, surgical success rate, and postoperative complications.

**Results:**

Baseline mean IOP was 23.06 ± 0.72 mmHg with a median of 3 (interquartile range, IQR: 2–3) medications. Postoperative IOP significantly decreased at all follow-up points except at 1 week (*p* < 0.05). At 12 months, the mean IOP was 16.22 ± 0.76 mmHg with a median of 0 (IQR: 0–1) medications. Complete surgical success rates were 80.0% at 6 months and 79.8% at 12 months. Early postoperative complications included IOP spikes in 34.9% and clinically significant hyphema in 6.3% (all grade 1). Cyclodialysis was rare (1.1%), and no severe adverse events were reported.

**Conclusion:**

3T appears to be a safe and effective surgical option for POAG, providing sustained IOP reduction and medication burden relief with a low incidence of postoperative complications. Larger, controlled studies with longer follow-up are warranted to further validate these findings.

## Introduction

Primary open-angle glaucoma (POAG) is the most common subtype of glaucoma worldwide, characterized by an insidious onset and irreversible vision loss (1). Sustained reduction of intraocular pressure (IOP) remains the only proven strategy to slow the progression of POAG (2). While medications and laser therapies are often effective in early-stage patients, surgical intervention is required for individuals with poor adherence, insufficient pharmacologic response, or rapid disease progression (3). Traditional filtering surgeries, such as trabeculectomy, are effective in lowering IOP but are associated with significant tissue disruption, a high risk of complications, and long-term failure due to bleb-related scarring (4). Therefore, there is an urgent clinical need for a safer, less invasive, and more sustainable surgical alternative.

Minimally invasive glaucoma surgery (MIGS) has emerged in recent years as a valuable complement to conventional filtering procedures. MIGS aims to restore physiological aqueous humor outflow via ab interno (from within the anterior chamber) or ab externo (from the ocular surface) approaches, with minimal tissue disruption, faster recovery, and a favorable safety profile (5). Common MIGS techniques include gonioscopy-assisted transluminal trabeculotomy (GATT), canaloplasty (CP), and ab interno canaloplasty (ABiC) (6, 7). GATT enables 360° trabeculotomy but may result in a relatively high incidence of hyphema and anterior chamber inflammation (5). CP is well-tolerated but achieves only modest IOP reduction due to limited outflow tract dilation (6). ABiC preserves the trabecular meshwork but may be compromised by postoperative collapse or reclosure of Schlemm’s canal (6). Consequently, none of the existing MIGS techniques fully balance efficacy, anatomical preservation, and safety, highlighting the need for further refinement.

**Figure 1:**
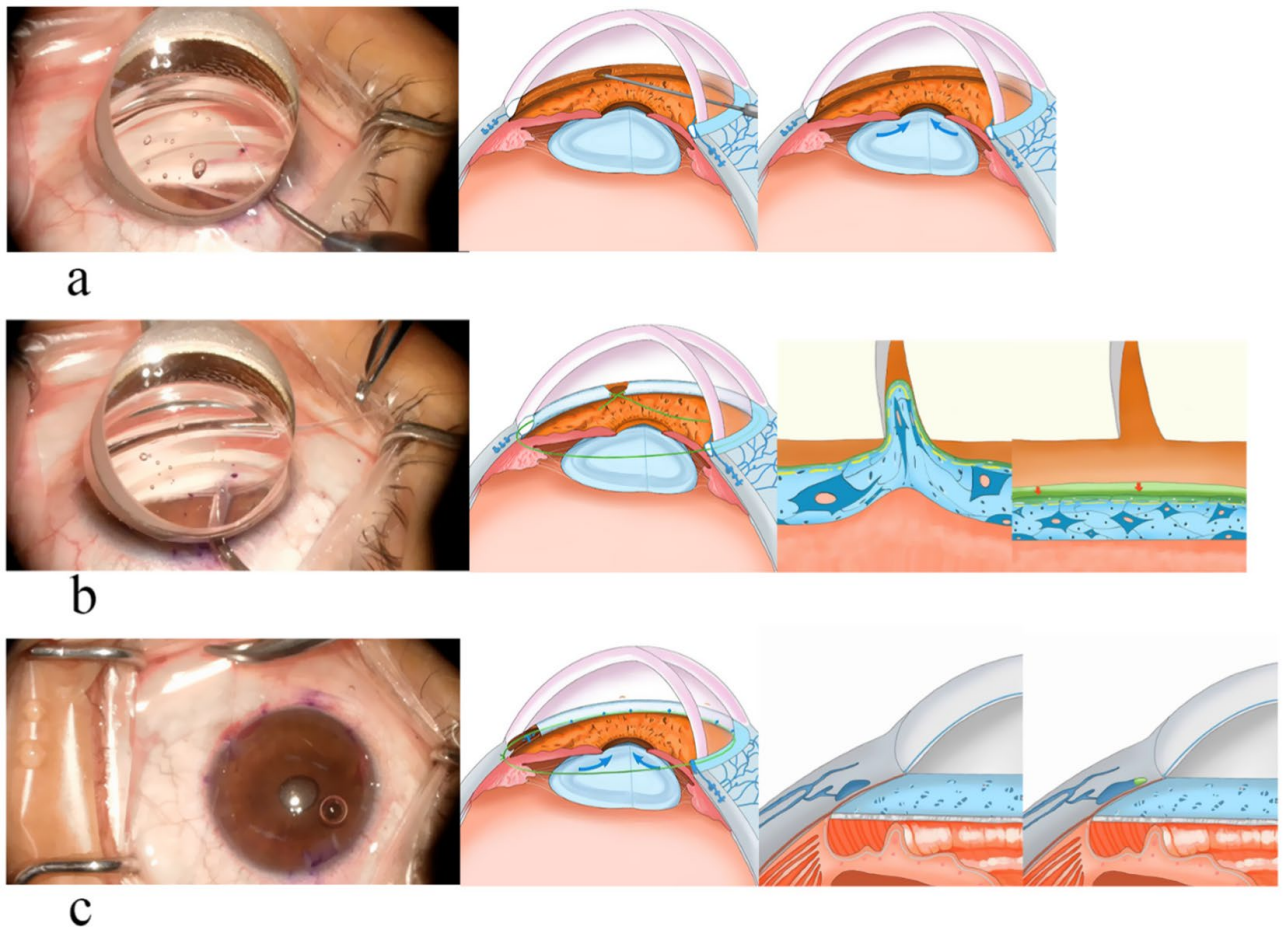
Mechanism of IOP lowering by 3T. **(a)** Trabeculotome: the trabecular meshwork was fenestrated, and the aqueous humor entered Schlemm’s canal through the fenestrated window. **(b)** Tunneling: the viscoelastic agent was used to expand, relieve the hernia of the trabecular meshwork, and expand the inlet of the collecting tube. **(c)** Trabeculoplasty: tension sutures were used for long-term dilation of Schlemm’s canal. These figures were created by the authors and are based on actual surgical steps performed at our institution. No copyrighted material or third-party content was used.

Trabeculotome tunneling trabeculoplasty (3T) is a novel ab interno MIGS procedure designed to integrate the advantages of existing approaches while addressing their limitations. The 3T technique consists of three key components (Figure 1): (1) trabecular window creation, which facilitates aqueous entry into Schlemm’s canal while preserving the biomechanical pump function of the remaining trabecular meshwork; (2) viscodilation of Schlemm’s canal and collector channel openings to relieve collapse and reduce trabecular herniation; and (3) long-term placement of a tensioning suture within Schlemm’s canal to maintain canal patency and enhance distal outflow. Preliminary studies suggest that 3T surgery can significantly lower IOP, reduce medication dependence, and maintain a low complication rate, indicating its potential as a promising MIGS alternative.

The present study aims to evaluate the early surgical outcomes and safety profile of the 3T procedure in patients with POAG and to provide evidence supporting its clinical application and future investigation.

## Subjects and methods

### Ethical approval

This study was a retrospective observational study with prospective follow-up, conducted at the Department of Ophthalmology, Chengdu First People’s Hospital. Clinical data were initially collected as part of routine care for patients undergoing 3T surgery for primary open-angle glaucoma (POAG) between December 2022 and October 2023. These data were retrospectively reviewed after a formal research protocol was developed, and ethics approval was obtained in early 2024 (Approval No. 2024XJS005).

Following ethics approval, follow-up for eligible patients was prospectively extended using standardized procedures to ensure completeness of outcome data up to 12 months. Additional patients treated between November 2023 and June 2024 were enrolled and followed prospectively under the approved protocol.

Although our hospital participated in a registered multicenter trial comparing 3T and GATT (ChiCTR2200066650), this analysis included only patients who received 3T surgery and were not enrolled in the comparative study. No randomization or experimental interventions were involved.

All patients provided written informed consent prior to surgery for their anonymized clinical data to be used for academic research.

All illustrations and intraoperative images presented in this article are original and created by the authors. Schematic diagrams were designed by the research team based on real surgical procedures, and intraoperative images were edited from anonymized surgical videos recorded at Chengdu First People’s Hospital. No patient-identifiable information or third-party copyrighted content is included.

### Subjects and preoperative assessments

All patients were diagnosed with POAG, defined as pathologically elevated IOP (peak IOP >21 mmHg within 24 h), accompanied by glaucomatous optic nerve damage and/or glaucomatous visual field defects, an open anterior chamber angle, and the exclusion of other factors that may cause elevated IOP. Inclusion criteria were as follows: (1) patients aged between 18 and 75 years at the time of signing the informed consent form, regardless of gender; (2) patients diagnosed with POAG, with IOP >21 mmHg despite maximum tolerated medical therapy, and a clear surgical indication for glaucoma intervention; (3) patients with no history of glaucoma surgery or only prior ocular procedures with confirmed intact Schlemm’s canal based on gonioscopy and imaging (ultrasound biomicroscopy or anterior segment optical coherence tomography). Acceptable procedures included selective laser trabeculoplasty (SLT). Patients with prior GATT, iStent, or trabectome were generally excluded, unless imaging clearly demonstrated an undamaged Schlemm’s canal; and (4) voluntary acceptance of surgery and the ability to cooperate with all required preoperative and postoperative examinations. Patients were required to meet all of the above criteria to be eligible for inclusion in the study.

**Figure 2:**
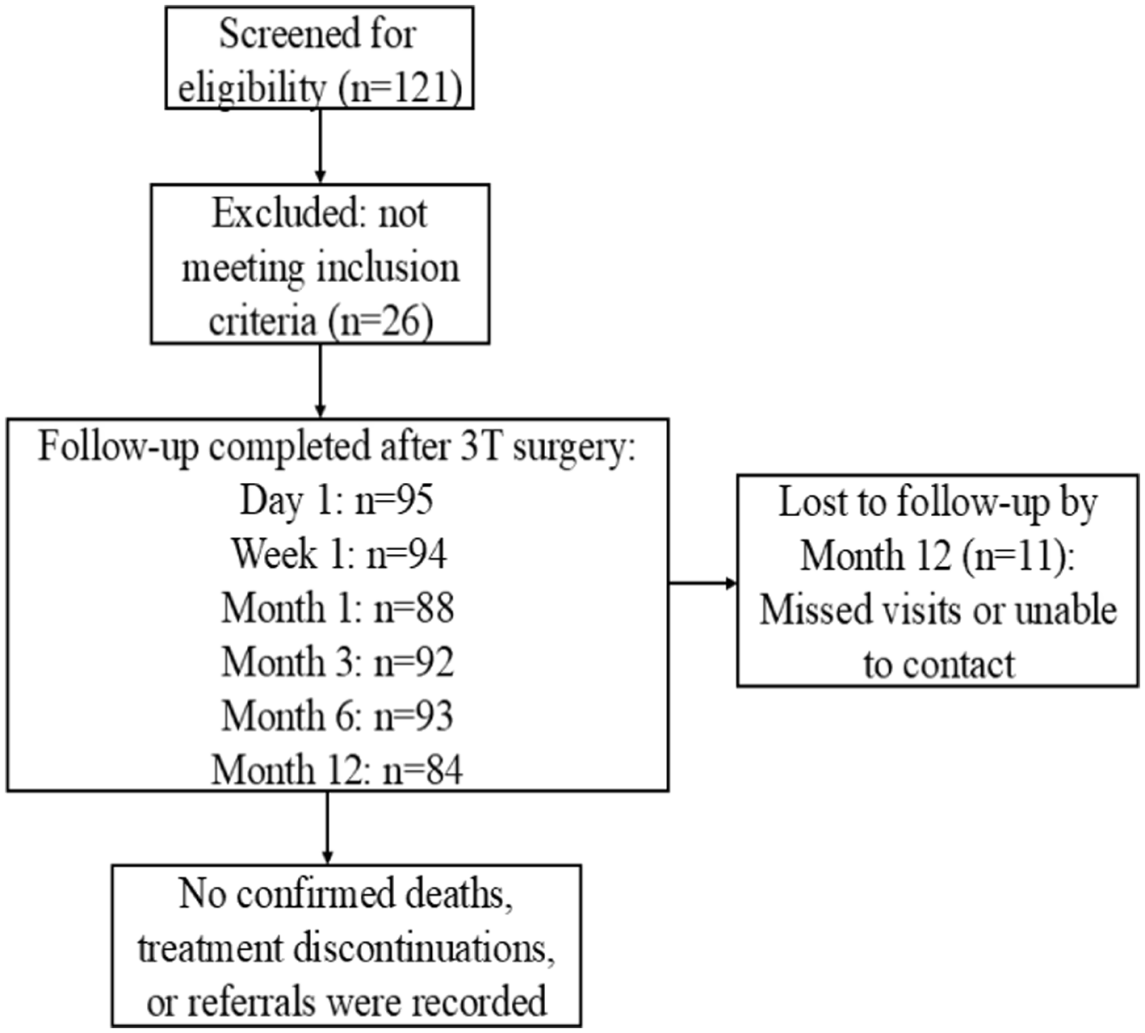
Patient flow diagram for the 3T surgery cohort.

Exclusion criteria were as follows: (1) patients with a history of previous ocular trauma or surgeries that may have disrupted the integrity of Schlemm’s canal; (2) patients in whom the anterior chamber angle could not be clearly visualized due to corneal opacity or who had abnormal anterior chamber angle structures; (3) patients with ocular or systemic conditions other than glaucoma, such as thyroid-associated ophthalmopathy, that may affect IOP; (4) patients with conditions that could affect the visual field, such as neurological disorders or intracranial tumors; (5) patients with severe heart or lung diseases, or advanced cancer, who were unable to tolerate ophthalmic surgery; (6) patients who were unable to complete all required preoperative and postoperative examinations; (7) pregnant or lactating women; and (8) any other conditions deemed unsuitable by the investigator. Patients who met any of the above criteria were excluded from the study. The detailed flowchart is shown in Figure 2.

All participants received a comprehensive ophthalmic examination within 1 week before the surgery, which included best-corrected visual acuity (BCVA), IOP measurement with non-contact jet tonometer, slit-lamp biomicroscopy, gonioscopy, ultrasound biomicroscopy, non-mydriatic fundus photography, the Humphrey 24-2 SITA standard visual field test, and retinal nerve fiber thickness assessment by optical coherence tomography.

### Surgical techniques

**Figure 3:**
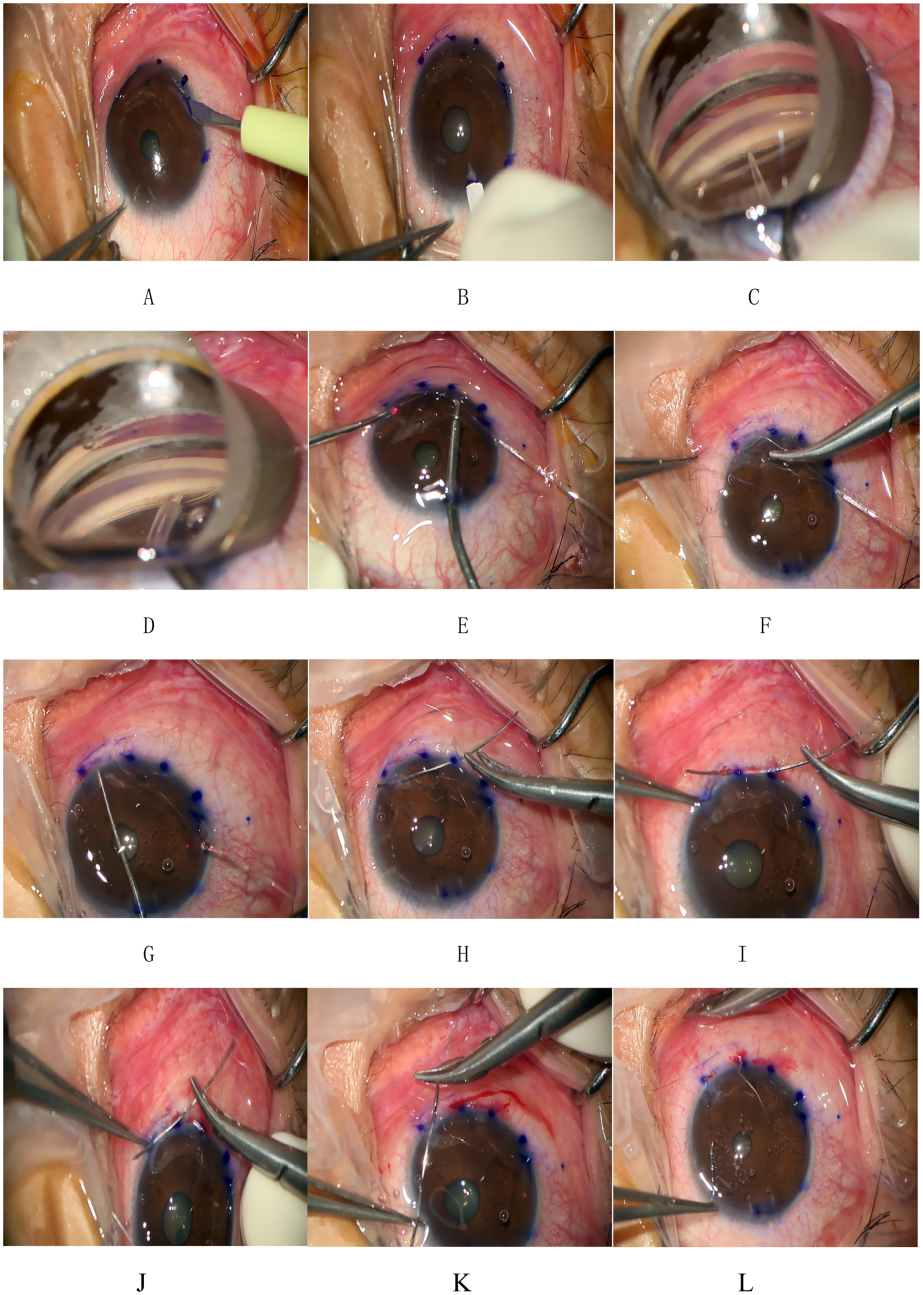
Procedure of 3T. **(A,B)** Make an auxiliary incision and a main incision at the transparent corneal margin. **(C)** Pre-position a microcatheter in the anterior chamber at the auxiliary incision and then incise the trabecular meshwork and the inner wall of Schlemm’s canal by approximately 2 mm under direct visualization using a gonioscope. **(D)** Insert the tip end of the microcatheter through the incision into Schlemm’s canal and advance it forward. After advancing counterclockwise for 360°, pass the tip end of the microcatheter out of the other end of Schlemm’s canal. **(E)** Make a transparent corneal limbus auxiliary incision at the end of the microcatheter and use microcapsular forceps to guide the tip of the microcatheter from the auxiliary incision to outside the eye. **(F)** Tie one end of a 10-0 polypropylene suture with a needle at the tip of the microcatheter. **(G)** Slowly withdraw the microcatheter from Schlemm’s canal and the eye. Cut the suture where it is tied to the microcatheter to separate it from the microcatheter. **(H)** Insert the large curved needle at the other end of the suture from the auxiliary incision into the anterior chamber and pass it through the eye 1 mm behind the corresponding corneal limbus at the point where the microcatheter exits Schlemm’s canal. **(I–K)** The thread with needle was threaded along the corneal rim between the layers through the clear corneal rim at the incision and into the anterior chamber and then through any corneal rim out of the eye and the excess suture with needle was cut. **(L)** The suture was tied at the clear corneal rim. These figures were created by the authors and are based on actual surgical steps performed at our institution. No copyrighted material or third-party content was used.

This surgery was pioneered by Dr. Ningli Wang. The operation method used in this study was improved by Professor Liuzhi Zeng. All patients were treated with 3T by the same experienced chief physician. The procedures are listed below (Figures 1, 3): (1) Routine disinfection, draping, and anesthesia. (2) Use a 15° surgical knife to make an auxiliary incision above the nose (left eye) or below the temporal region (right eye) at the transparent corneal margin and make a main incision of 1.8 mm in the superior temporal transparent corneal margin. (3). Pre-position a microcatheter in the anterior chamber at the auxiliary incision. (4) With gonioscope assistance, incise the nasal trabecular meshwork and the inner wall of Schlemm’s canal by approximately 2 mm and advance the microcatheter circumferentially through Schlemm’s canal. During the advancement, viscoelastic substance was injected approximately every 60° to 90° (i.e., every 2 to 3 clock-hour positions) to dilate the canal. After advancing counterclockwise for 360°, pass the tip of the microcatheter out of the other end of Schlemm’s canal. Make a transparent corneal limbus auxiliary incision at the end of the microcatheter, and guide its tip from the auxiliary incision to outside the eye. (5) Tie one end of a 10-0 polypropylene suture with a needle approximately 1–2 mm behind the tip of the microcatheter. Use intraocular forceps to retract the tip of the microcatheter into the anterior chamber. (6) After securing the microcatheter inserted into Schlemm’s canal with a Y-shaped positioning hook, slowly withdraw the microcatheter from Schlemm’s canal and the eye using external forceps. Cut the suture where it is tied to the microcatheter to separate it from the microcatheter. (7) Insert the large curved needle attached to the other end of the suture from the auxiliary incision into the anterior chamber, passing it through the eye 1 mm behind the corresponding corneal limbus at the point where the microcatheter exits Schlemm’s canal after entering the anterior chamber from the auxiliary incision. (8) Thread the needle and suture along the corneal rim between the layers through the clear corneal incision into the anterior chamber, then pass it through the corneal rim out of the eye, and cut the excess suture with the needle. (9) Hook the sutures at each end with a micro crochet hook through the auxiliary incision. (10) Expand the openings with viscoelastic substance under double-sided gonioscopic mirrors. (11) Flush the anterior chamber to remove viscoelastic and lower intraocular pressure to T-1 (indicating a slightly reduced IOP). (12) Tie the suture at the clear corneal rim, pre-cut the thread leaving a long knot, and flush the anterior chamber under pressure. (13) Trim the wire knot, hydrate the incision, and restore the anterior chamber. (14) Inject gas into the anterior chamber to raise the IOP to T + 1 (indicating a slightly elevated IOP), and complete the operation.

### Postoperative follow-up and outcome measures

Patients were followed up on the first day after the surgery and at the following time points (1 week, 1 month, 3 months, 6 months, and 12 months). Preoperative and postoperative evaluations included all baseline examinations and the number of glaucoma medications, intraoperative and postoperative complications, and additional interventions. A gonioscope was used to observe the postoperative angle. Complete success was defined as a reduction in IOP of ≥30% after surgery and no increase in the use of antiglaucoma medications, or IOP ≤21 mmHg without antiglaucoma medications. Qualified success was defined as postoperative IOP no higher than preoperative IOP and the reduction of at least two types of antiglaucoma medications. Considering the possibility of IOP spike in the early postoperative period, 6 months after surgery was used as the time point to judge the effectiveness of surgery. An IOP spike was defined as an IOP greater than or equal to 30 mmHg.

### Statistical analysis

All statistical analyses were performed using SPSS version 26.0 (IBM Corp., Armonk, NY, United States), with a two-sided *p*-value <0.05 considered statistically significant.

Continuous variables were tested for normality using the Shapiro–Wilk test and presented as mean ± standard deviation (SD) for normally distributed data, or as median with interquartile range (IQR) otherwise. Categorical variables were reported as counts and percentages.

IOP changes over time were analyzed using a linear mixed-effects model (LMM), with follow-up time and surgical laterality (unilateral vs. bilateral) as fixed effects and patient ID as a random effect to account for intra-subject correlation. An interaction term (time × laterality) was included to assess differences in IOP trajectories between the groups. To visualize overall IOP trends, repeated measures analysis of variance (RM-ANOVA) with Bonferroni correction was additionally performed.

The number of IOP-lowering medications at each follow-up point was compared with baseline using the Wilcoxon signed-rank test. Surgical success rates were analyzed using the chi-square test, including subgroup comparisons between eyes with and without early postoperative complications.

Factors associated with complete surgical success at 12 months were evaluated using multivariate logistic regression, including sex, baseline IOP, number of medications, and trabecular angle as independent variables. Model fit was assessed using the Hosmer–Lemeshow test.

Due to non-normal distribution and uneven group sizes, the Kruskal–Wallis test was used to examine the association between trabecular window size and IOP reduction at 12 months.

Missing data were not imputed; all analyses were based on available cases. Of the 95 eyes enrolled, 84 (88.4%) completed the 12-month follow-up.

### Sample size estimation

Sample size estimation was based on two primary outcomes: postoperative IOP reduction and surgical success rate. Drawing from previous studies on Schlemm’s canal-based MIGS (e.g., GATT), we assumed a mean IOP reduction of 5 mmHg with a standard deviation of 6 mmHg. Using a paired *t*-test (*α* = 0.05, power = 80%), the minimum required sample size was estimated to be 15 eyes. For surgical success, we assumed a complete success rate of 70% and used a one-sample proportion test against a reference rate of 50%, which yielded a required sample size of 40 eyes.

A total of 95 eyes were included in this study, exceeding the minimum sample size requirements and providing adequate statistical power to support the primary analyses. Sample size calculations were performed using G*Power 3.1 and Statulator,^1^ with assumptions based on prior literature (8–10).

## Results

The study included 95 eyes from 72 patients with POAG. The average age of the patients (23 women and 49 men) who underwent 3T was 47.34 ± 17.44 years (range: 18–75). Demographic and clinical characteristics of these patients are summarized in Table 1. The preoperative IOP in eyes that successfully underwent 3T was 23.06 ± 0.72 mmHg while receiving a median of 3 IOP-lowering medications (interquartile range: 2–3; range: 1–4). Prior to surgery, 81.05% (77 eyes) were treated with fewer than four types of IOP-lowering medications, while 18.95% (18 eyes) received four or more. The preoperative mean deviation (MD) on automated perimetry was −16.88 dB (IQR: −27.74 to −8.41; range: −37.81 to −1.71). Preoperative BCVA in logMAR was 0.30 (IQR: 0.10–1.00). Of the 95 eyes enrolled at baseline, 84 eyes (88.4%) completed the 12-month follow-up. Eleven eyes were lost to follow-up due to non-attendance at scheduled visits and inability to contact by phone.

**Table 1:** Demographic and clinical characteristics of these patients with 3T.

Variable	*N*	Mean values
No. of eyes/patients	95/72	
Age, years, range		47.34 ± 17.44, 18–75
Sex, male/female	49/23	
OD/OS	44/51	
Preoperative IOP (mmHg)		23.06 ± 0.72
BCVA, logMAR (mean ± SD)		0.30102 (0.09691, 1)
Preoperative MD, dB		−16.88 (−27.74, −8.41) (−37.81 to −1.71 dB)
Preoperative antiglaucoma medications (mean ± SD)		3 (2, 3)
Number of medications, *n* (%)	3 (2, 3)	
<4	77 (81.05%)	
≥4	18 (18.95%)	

Data are expressed as the mean ± SD or as the absolute value (percentage) as appropriate. BCVA, best-corrected visual acuity; IOP, intraocular pressure; logMAR, logarithmic minimum angle of resolution; SD, standard deviation.

### IOP changes over time

An LMM was used to evaluate IOP changes over time while accounting for repeated measures and the inclusion of bilateral cases. Time and surgical laterality (unilateral vs. bilateral) were modeled as fixed effects and patient ID as a random effect. The analysis revealed a significant main effect of time on IOP (*p* < 0.001), as well as a significant interaction between time and laterality (*F* = 2.835, *p* = 0.011), indicating that the temporal IOP pattern differed between unilateral and bilateral cases (Table 2).

**Table 2:** Tests of fixed effects in the linear mixed-effects model for IOP.

Source	df (numerator)	df (denominator)	*F*	*p*-value
Intercept	1	252.444	4735.081	<0.001^**^
Eye (laterality)	1	252.444	0.292	0.589
Timepoint	6	192.557	24.668	<0.001^**^
Eye × timepoint	6	192.557	2.835	0.011^*^

Dependent variable: intraocular pressure (IOP) ^*^p < 0.05 and ^**^p < 0.01.

Specifically, unilateral cases had higher baseline IOP (25.20 mmHg) than bilateral cases (20.91 mmHg). Both groups showed substantial IOP reduction at day 1, but a sharper transient increase was noted at week 1 in bilateral cases (27.44 mmHg vs. 23.47 mmHg). From month 1 onward, IOP remained stable and comparable between the two groups (approximately 16–17 mmHg), confirming long-term surgical effectiveness regardless of laterality (Table 3).

**Table 3:** Estimated marginal means of IOP over time in unilateral and bilateral surgeries.

Laterality	Time point	Mean (mmHg)	SE	95% CI lower	95% CI upper
Unilateral	Baseline	25.204	0.997	23.246	27.162
	1 day	16.245	0.997	14.287	18.203
	1 week	23.469	0.997	21.511	25.428
	1 month	17.867	1.041	15.823	19.910
	3 months	16.638	1.018	14.639	18.638
	6 months	15.917	1.008	13.933	17.901
	12 months	15.981	1.008	14.538	17.424
Bilateral	Baseline	20.913	1.029	18.896	22.934
	1 day	15.196	1.029	13.180	17.213
	1 week	27.444	1.029	25.428	29.460
	1 month	19.176	1.074	17.063	21.289
	3 months	16.396	1.051	14.331	18.461
	6 months	16.644	1.041	14.604	18.684
	12 months	15.301	1.041	13.664	16.938

Dependent variable: intraocular pressure (IOP).

**Figure 4:**
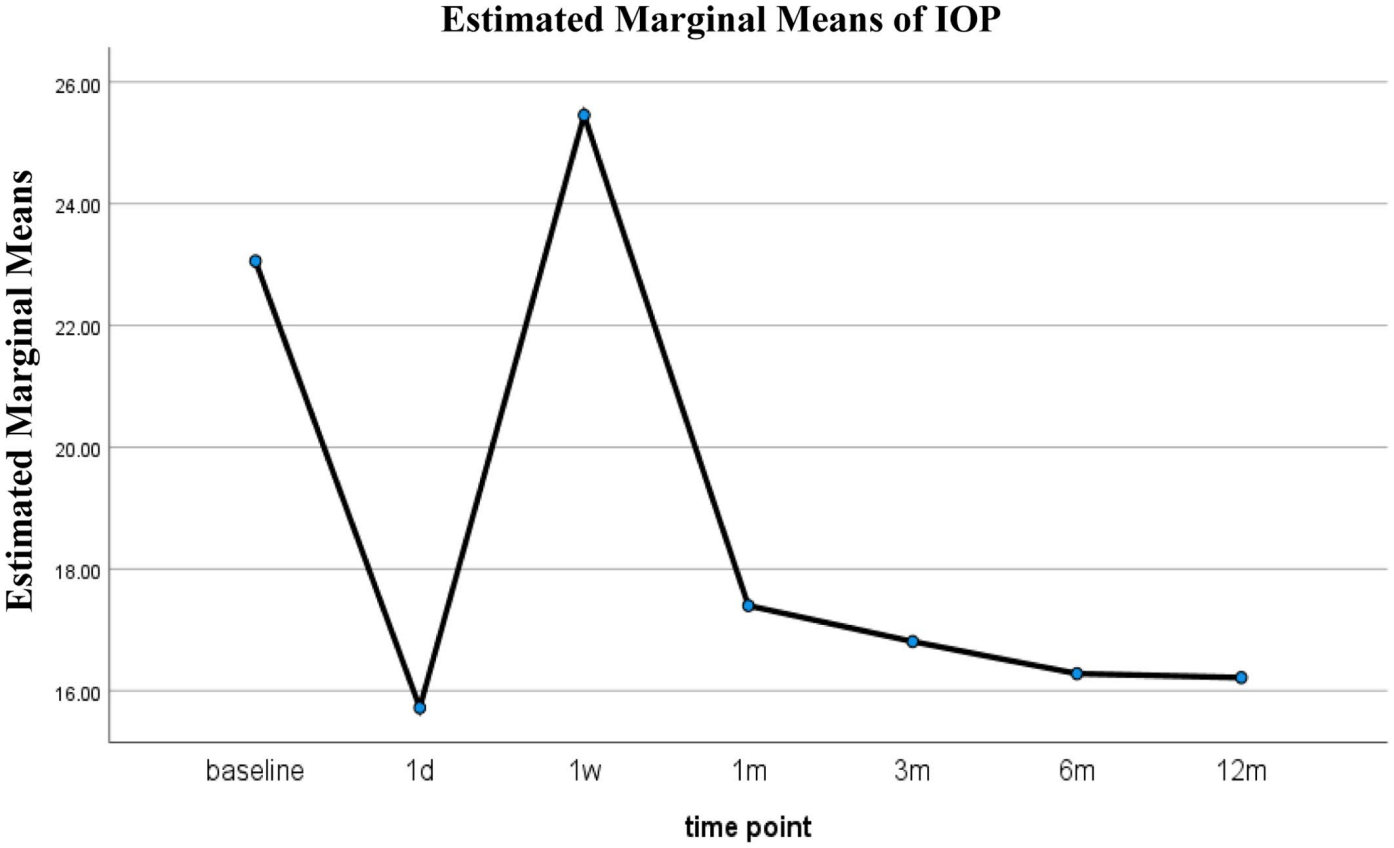
Estimated IOP over time after 3T.

To complement the model-based analysis and visualize the overall trend of IOP over time, RM-ANOVA was also performed. This analysis also revealed a significant effect of time (*p* < 0.001). Bonferroni-corrected pairwise comparisons showed that IOP decreased significantly from baseline at all follow-up time points except for week 1 (*p* = 0.603) (Table 4). The estimated marginal means (±standard error, SE) of IOP were 23.06 ± 0.72 mmHg at baseline, 15.72 ± 0.72 mmHg at day 1, 25.46 ± 0.72 mmHg at week 1, 17.40 ± 0.74 mmHg at month 1, 16.81 ± 0.73 mmHg at month 3, 16.28 ± 0.72 mmHg at month 6, and 16.22 ± 0.76 mmHg at month 12 (Table 5 and Figure 4).

**Table 4:** Pairwise comparisons of estimated marginal means of IOP across time points (Bonferroni-adjusted).

(I) Time point	(J) Time point	Mean diff. (I − J)	SE	*p*-value^a^	95% CI lower	95% CI upper
Baseline	1 day	7.389^*^	1.022	<0.001^**^	4.273	10.506
	1 week	–2.246	1.024	0.603	–5.371	0.879
	1 month	5.717^*^	1.042	<0.001^**^	2.540	8.895
	3 months	6.322^*^	1.030	<0.001^**^	3.180	9.464
	6 months	6.857^*^	1.027	<0.001^**^	3.724	9.991
	12 months	6.900^*^	1.055	<0.001^**^	3.683	10.117

^*^p < 0.05 and ^**^p < 0.01.

a Adjustment for multiple comparisons: Bonferroni method.

**Table 5:** Estimated marginal means of IOP at each postoperative time point.

Time point	Mean IOP (mmHg)	Standard error	95% Confidence interval (lower)	95% Confidence interval (upper)
Baseline	23.059	0.717	21.652	24.466
1 day	15.720	0.717	14.313	17.127
1 week	25.457	0.721	24.042	26.872
1 month	17.398	0.744	15.937	18.860
3 months	16.808	0.728	15.379	18.237
6 months	16.281	0.724	14.858	17.703
12 months	16.216	0.762	14.720	17.712

### Medication use

The number of IOP-lowering medications significantly decreased at all postoperative time points compared to baseline (*p* < 0.001 for all comparisons, Wilcoxon signed-rank test). At baseline, the median number of medications was 3 (IQR: 2–3), which dropped to 0 (0–0) on postoperative day 1. The median remained low during follow-up: 0 (0–2) at week 1, 0 (0–1) at months 1 and 3, 0 (0–0.5) at month 6, and 0 (0–1) at month 12. These results indicate a sustained reduction in medication burden following surgery, as shown in Table 6.

**Table 6:** Changes in the number of IOP-lowering medications at each postoperative time point compared with baseline.

Time point	*N*	Median (IQR) of medications	*p*-value vs. baseline
Baseline	95	3 (2–3)	—
1 day	95	0 (0–0)	<0.001
1 week	94	0 (0–2)	<0.001
1 month	88	0 (0–1)	<0.001
3 months	92	0 (0–1)	<0.001
6 months	93	0 (0–0.5)	<0.001
12 months	84	0 (0–1)	<0.001

*p*-values were obtained using the Wilcoxon signed-rank test, with each postoperative time point compared to the baseline using paired analyses.

### Surgical success and postoperative complications

The procedure achieved consistently high surgical success rates, with 90.0% at 6 months and 86.9% at 12 months, demonstrating sustained efficacy over the first postoperative year. Complete success was achieved in 80.0 and 79.8% of eyes at 6 and 12 months, respectively, as shown in Table 7.

**Table 7:** Analysis of surgical success rate.

Time point	Complete success rate	Qualified success rate	Surgical success rate
6 months	80.0%	10.0%	90.0%
12 months	79.8%	7.1%	86.9%

**Figure 5:**
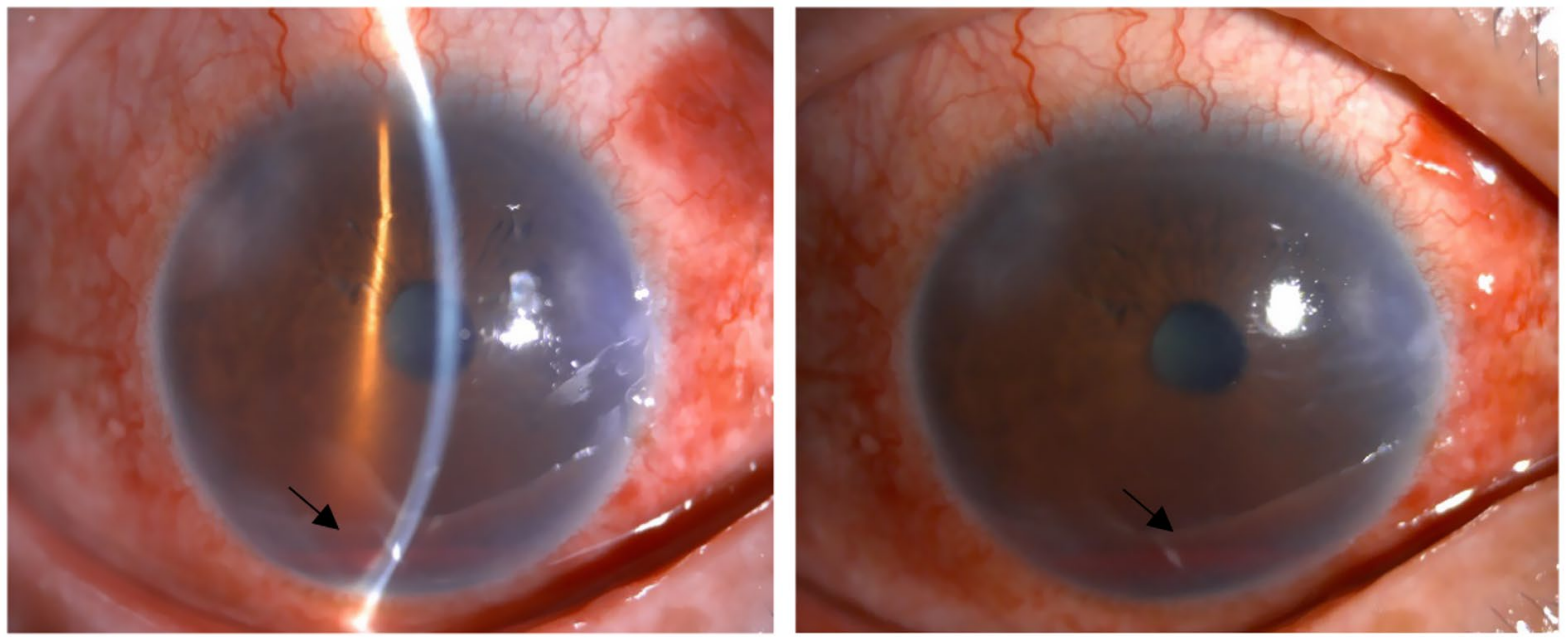
Early postoperative hyphema. The two black arrows indicate postoperative hyphema. Above are from the same eye.

**Figure 6:**
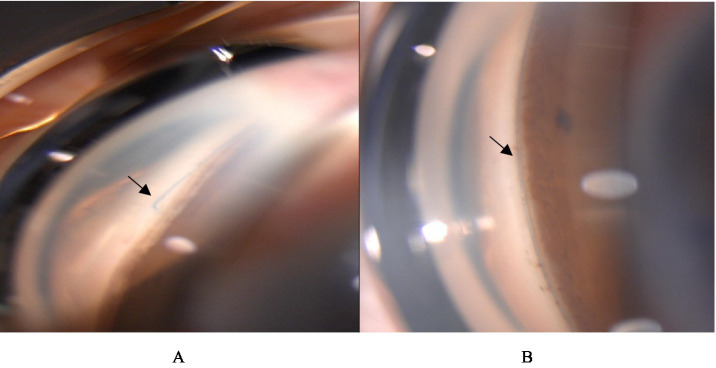
Gonioscopy was performed 1 month after surgery. **(A)** The black arrow indicates the site where the suture comes out of the opening window of the trabecular meshwork. **(B)** The black arrow indicates that the suture is located in Schlemm’s channel. Above are from the same eye.

There were few surgical complications during the 12-month follow-up. Only 6.3% of eyes experienced clinically significant hyphema, which resolved spontaneously within 5 days after surgery. All hyphema cases observed were classified as grade 1 (Figure 5). According to the European Glaucoma Society guidelines, hyphema is categorized into two grades: grade 1, where blood fills less than one-third of the anterior chamber, and grade 2, characterized by complete filling of the anterior chamber with blood. Notably, only one eye developed hyphema that did not resolve spontaneously and required anterior chamber irrigation 10 days following the 3T procedure. Transient IOP elevation (30–60 mmHg) occurred in 34.9% of eyes, with a median duration of 7.55 ± 4.84 days (range, 1–21 days). The mean time to onset of the IOP spike was 8.85 ± 5.75 days postoperatively (range, 3–30 days). When IOP spikes occurred after surgery, we would actively provide IOP reduction treatment. Initially, carbonic anhydrase inhibitors were selected for the treatment to lower IOP. If the IOP was elevated at this time, *α*-receptor agonists and β-receptor blockers were continued. If the maximum dose of eye drops still cannot reduce IOP, systemic antiglaucoma drugs, such as oral acetazolamide tablets and intravenous mannitol, are recommended, but it is necessary to pay attention to the patient’s systemic contraindications. In refractory cases, anterior chamber paracentesis or additional surgical intervention was performed. Notably, five eyes eventually required secondary surgical procedures due to persistent IOP elevation despite maximal medical therapy—two eyes at 2 months postoperatively, two at 1 month, and one at 4 months. The complication rate of cyclodialysis was also very low, only 1.1%. Once it occurred, the eyes were treated using a pressure bandage, as shown in Table 8. No other intraoperative or postoperative complications were observed. The postoperative anterior segment condition of the eyes was gratifying, and there was almost no inflammatory reaction, which truly demonstrated the minimally invasive characteristics of 3T surgery. Gonioscopy was performed 1 month after surgery, and the position of the sutures could be clearly seen (Figure 6).

**Table 8:** Intra- and postoperative complications according to frequency.

Complications	*N*
IOP spike	34.9% (30–60 mmHg)
Hyphema	6.3%
Cyclodialysis	1.1%

Among the 84 eyes with complete 12-month follow-up, 41 (48.8%) experienced early postoperative complications. The success rate was 82.9% (34/41) in the complication group and 90.7% (39/43) in the non-complication group. The difference was not statistically significant (chi-square = 1.114, *p* = 0.291).

### Factors associated with surgical success

A multivariate logistic regression was conducted to explore baseline and perioperative factors associated with surgical success at 12 months, including sex, number of medications, preoperative IOP, and trabecular angle. The model showed good fit (Hosmer–Lemeshow *p* = 0.129) but was not statistically significant overall (omnibus *p* = 0.187), with low explanatory power (Nagelkerke’s pseudo coefficient of determination, *R*^2^ = 0.112). None of the variables reached significance, although sex showed a trend toward association (*p* = 0.082). The model’s overall accuracy was 79.8%, but it failed to correctly classify any failure cases, likely due to class imbalance and limited sample size. These results suggest limited predictive value of the included variables (Table 1 of the attachment). Further studies with larger, balanced samples and additional factors are needed.

### Association between trabecular window size and IOP reduction

To evaluate the association between the extent of the trabecular window and the magnitude of IOP reduction at 12 months, we performed a Kruskal–Wallis test across different window size groups. The test showed no statistically significant difference in IOP reduction among groups (*p* = 0.388). The data were highly imbalanced, with 70 eyes in the 60° group and very few cases in other angle groups (e.g., 45°, 75°, 90°, 120°, and 180°). These results suggest that the extent of trabecular opening may not significantly influence postoperative IOP reduction, although further studies with more balanced group sizes are needed to confirm this.

Additionally, visual field data (MD values) were available for only 37% of eyes at 12 months. Due to the limited sample size and potential follow-up bias, no statistical analysis was performed. Similarly, BCVA data were available for only 36% of eyes, among which most showed stable or minimally changed visual acuity compared to the baseline. Given the small sample size, formal statistical testing was not conducted. Nevertheless, the available results suggest that most eyes maintained stable visual acuity, and no vision-threatening complications were observed.

## Discussion

Conventional trabeculectomy is associated with a high rate of complications (11–13), prompting the emergence of MIGS (14–17). Although current MIGS techniques, such as GATT and ABiC (18–22), offer improved safety profiles, limitations remain—particularly related to damage to the trabecular meshwork and the potential for Schlemm’s canal collapse (23). Thus, there is an ongoing need for surgical approaches that are both effective and minimally disruptive to the ocular outflow structures. 3T, developed by Professor Ningli Wang in 2022, integrates trabecular window creation, viscodilation, and tensioning suture placement to preserve trabecular meshwork function and maintain long-term patency of Schlemm’s canal (24). Preliminary evidence suggests that 3T not only significantly lowers IOP but also enhances the biomechanical aqueous outflow pump mechanism, supporting its potential as a promising MIGS alternative.

In our study, 84 eyes completed the 12-month follow-up, yielding a completion rate of 88.4%, which is generally acceptable in surgical research. Approximately 11.6% of eyes were lost to follow-up, primarily due to missed clinic visits or inability to contact the patients. Although the proportion of missing data was relatively low and the missingness was assumed to be random, a small risk of follow-up bias cannot be entirely excluded and should be acknowledged.

Compared with most previously published studies on MIGS, our cohort exhibited more advanced disease at baseline, with a median visual field MD of −16.88 dB and a subset of patients demonstrating marked visual acuity reduction (logMAR BCVA up to 1.00). Notably, these characteristics were not the result of selective recruitment. Instead, patients were enrolled consecutively based on eligibility for the 3T procedure, reflecting typical clinical presentations in a tertiary glaucoma center. This suggests that 3T may be a viable surgical option not only for early- to moderate-stage POAG but also for patients with more advanced disease, including those who are not ideal candidates for traditional filtering surgery. The broader range of disease severity captured in this study highlights the real-world applicability of the 3T procedure and should be taken into account when interpreting postoperative outcomes and comparing them with those of prior studies focused on earlier-stage disease.

Our results demonstrated a sustained and clinically meaningful reduction in IOP after 3T surgery. At 12 months, the mean IOP decreased by 6.6 mmHg from baseline, corresponding to a 29.2% reduction. This was accompanied by a significant decrease in the use of IOP-lowering medications—from 100% of eyes at baseline to 24% at 12 months—highlighting the procedure’s effectiveness in reducing both IOP and medication burden. Notably, both RM-ANOVA and LMM analyses confirmed that the IOP remained stable from 1 month onward, with no significant differences between unilateral and bilateral cases in the long term. These findings support the durable pressure-lowering efficacy of 3T surgery.

The surgical success rates in our study were high, with overall success at 90.0 and 86.9% at 6 and 12 months, respectively. Complete success was achieved in 80.0 and 79.8% of eyes, and qualified success was achieved in 10 and 7.1%, respectively. These rates appear higher than those reported for GATT in previous studies. One study found that the 12-month rate of complete surgical success was 75% in the GATT group for POAG patients (18). Another study found that the complete and qualified success rates were 70.8 and 81.2% at 12 months, and 58.6 and 81.2% at 18 months, respectively, of GATT for juvenile-onset POAG (25). Sang et al. (26) found that there was no significant difference in complete success rate and qualified success rate between 3T and GATT in the treatment of POAG at 3 months after surgery. Of course, because of the different criteria for defining the success rate of surgery, the success rate of surgery will also be different. However, the sample size included in the study, the duration of follow-up, race, and the proficiency of the surgical operator are also important factors (27–28). Nevertheless, these findings suggest that the 3T procedure provides relatively stable efficacy and may offer certain advantages.

The incidence of clinically significant hyphema in our cohort was low (6.3%), and all were grade 1, resolving spontaneously within 5 days except for one case. This rate is substantially lower than those reported for GATT. A recent randomized controlled trial by Yin et al. (18) reported an 87% hyphema rate following GATT in patients with POAG, compared to a significantly lower rate in the ABiC group. Similarly, other studies have reported hyphema rates ranging from 12.5% to over 60%, depending on surgical technique and extent of trabecular disruption (29–31). The more localized trabecular window (∼60°), absence of full-thickness trabeculotomy, and preservation of trabecular meshwork integrity in 3T may account for the reduced risk of reflux bleeding. Furthermore, the retained trabecular tissue may act as a tamponade, minimizing blood reflux during the early postoperative period. Cyclodialysis was rare (1.1%), whereas GATT showed significantly higher rates. This likely reflects the more conservative dissection and reduced traction on the ciliary body inherent to the 3T approach, supporting its favorable safety profile.

In our cohort, the postoperative IOP rebound rate after 3T surgery was 34.9%. This is comparable to the rebound rate reported by Sang et al. (24), who found a 35.0% IOP rebound rate in patients undergoing 360° GATT. To date, the mechanism of IOP spike remains unclear. IOP elevation has been reported across various procedures involving Schlemm’s canal, and 3T is no exception. We consider that this phenomenon may be partly attributed to the intraoperative injection of viscoelastic agent during the passage of the microcatheter into and out of Schlemm’s canal. In addition, early mechanical stimulation from the suture left within Schlemm’s canal may also contribute to the IOP spike. Some studies have further suggested that disease severity and patient age may be potential risk factors for postoperative IOP spikes (19), which will be the focus of our subsequent research. Compared to GATT, the 3T procedure—featuring trabeculotome tunneling rather than a full circumferential incision—may cause less trauma to the trabecular meshwork and Schlemm’s canal, potentially resulting in a more stable IOP profile. However, further prospective studies with longer follow-up are necessary to validate this hypothesis and determine the long-term clinical advantages of 3T.

We utilized both RM-ANOVA and LMM to analyze IOP trajectories. The LMM model incorporated patient ID as a random effect and identified a significant interaction between time and laterality, suggesting differing IOP dynamics in the early postoperative period between unilateral and bilateral procedures. These differences converged over time, reinforcing the importance of early IOP monitoring and highlighting the stability of 3T outcomes across subgroups.

We also found that the extent of trabecular meshwork fenestration was not significantly correlated with IOP reduction at 12 months postoperatively. This finding may be partially influenced by the design of the 3T procedure itself, which typically involves a standardized trabecular meshwork fenestration of approximately 60°. In our study, 70 eyes underwent this standard approach, resulting in limited variability in the range of fenestration and restricting our ability to further analyze any potential dose–response relationship between the extent of incision and surgical efficacy. Although some studies have hypothesized that a broader trabeculotomy may enhance aqueous outflow, existing evidence on this relationship remains inconsistent. For instance, Grover et al. (32) and Seibold et al. (33) reported that the magnitude of IOP reduction does not necessarily increase linearly with the arc of trabecular incision, likely due to segmental aqueous flow and resistance at the level of the distal collector channels. Our findings are consistent with this view, suggesting that a localized and precise approach, such as that used in the 3T procedure, may still achieve effective IOP control.

Follow-up data demonstrated that this procedure offers promising efficacy and safety. Notably, the incidence of postoperative complications was low, indicating a favorable safety profile. However, several limitations of this study should be acknowledged. First, we did not include a control group, which limits the ability to make direct comparisons with other minimally invasive glaucoma surgeries or conventional filtering procedures (34). Second, we did not analyze pre- and postoperative corneal endothelial cell density and thus could not evaluate the potential impact of the surgery on the corneal endothelium (35). Third, although the follow-up duration was sufficient to assess early surgical outcomes, it was relatively short for a chronic and progressive disease such as glaucoma and may not fully reflect long-term efficacy and complication risks (36). Our logistic regression model showed limited predictive power, likely due to the small sample size and imbalance between success and failure groups. Additional perioperative variables and larger, balanced cohorts are warranted in future studies. In addition, we did not obtain morphological imaging of Schlemm’s canal and the trabecular meshwork before and after surgery—such as anterior segment optical coherence tomography—thereby lacking direct structural evidence to support the proposed surgical mechanism (37, 38). This is one of the key limitations of our study. In future studies, we plan to focus on this aspect by incorporating quantitative imaging analysis to better assess anatomical changes in Schlemm’s canal and the trabecular meshwork and thereby deepen our understanding of the mechanism of action of the 3T procedure. Therefore, future prospective, randomized controlled studies with larger sample sizes, longer follow-up durations, and comprehensive imaging and functional assessments are needed to fully validate the long-term efficacy and safety of the 3T procedure.

## Conclusion

3T appears to be a promising minimally invasive surgical technique for POAG, showing significant IOP reduction, reduced medication burden, and a favorable safety profile at 12 months postoperatively. These findings support the potential clinical value of 3T, while further studies with longer follow-up and comparative designs are warranted.

## Data Availability

The datasets presented in this study can be found in online repositories. The names of the repository/repositories and accession number(s) can be found in the article/Supplementary material.

## Footnotes

^1^ https://statulator.com

## Supplementary material

SUPPLEMENTARY VIDEO 1 Full procedural video of the 3T surgical technique as described in the manuscript, illustrating the step-by-step operative approach.
